# Comparison of the Translational Potential of Human Mesenchymal Progenitor Cells from Different Bone Entities for Autologous 3D Bioprinted Bone Grafts

**DOI:** 10.3390/ijms22020796

**Published:** 2021-01-14

**Authors:** Anna-Klara Amler, Patrick H. Dinkelborg, Domenic Schlauch, Jacob Spinnen, Stefan Stich, Roland Lauster, Michael Sittinger, Susanne Nahles, Max Heiland, Lutz Kloke, Carsten Rendenbach, Benedicta Beck-Broichsitter, Tilo Dehne

**Affiliations:** 1 Department of Medical Biotechnology, Technische Universität Berlin, 13355 Berlin, Germany; aka@cellbricks.com (A.-K.A.); D.schlauch@campus.tu-berlin.de (D.S.); roland.lauster@tu-berlin.de (R.L.); 2 Cellbricks GmbH, 13355 Berlin, Germany; lk@cellbricks.com; 3 Charité-Universitätsmedizin Berlin, Corporate Member of Freie Universität Berlin, Humboldt Universität zu Berlin, Department of Oral and Maxillofacial Surgery, and Berlin Institute of Health, 13353 Berlin, Germany; susanne.nahles@charite.de (S.N.); max.heiland@charite.de (M.H.); carsten.rendenbach@charite.de (C.R.); benedicta.beck-broichsitter@charite.de (B.B.-B.); 4 Charité-Universitätsmedizin Berlin, Corporate Member of Freie Universität Berlin, Humboldt Universität zu Berlin, Department of Rheumatology, and Berlin Institute of Health, 10117 Berlin, Germany; jacob.spinnen@charite.de (J.S.); stefan.stich@charite.de (S.S.); michael.sittinger@charite.de (M.S.); tilo.dehne@charite.de (T.D.)

**Keywords:** bioprinting, tissue engineering, gelatin methacrylate, regenerative medicine, segmental bone defect, mesenchymal progenitor cell, osteogenic differentiation, stereolithography, biomaterial

## Abstract

Reconstruction of segmental bone defects by autologous bone grafting is still the standard of care but presents challenges including anatomical availability and potential donor site morbidity. The process of 3D bioprinting, the application of 3D printing for direct fabrication of living tissue, opens new possibilities for highly personalized tissue implants, making it an appealing alternative to autologous bone grafts. One of the most crucial hurdles for the clinical application of 3D bioprinting is the choice of a suitable cell source, which should be minimally invasive, with high osteogenic potential, with fast, easy expansion. In this study, mesenchymal progenitor cells were isolated from clinically relevant human bone biopsy sites (explant cultures from alveolar bone, iliac crest and fibula; bone marrow aspirates; and periosteal bone shaving from the mastoid) and 3D bioprinted using projection-based stereolithography. Printed constructs were cultivated for 28 days and analyzed regarding their osteogenic potential by assessing viability, mineralization, and gene expression. While viability levels of all cell sources were comparable over the course of the cultivation, cells obtained by periosteal bone shaving showed higher mineralization of the print matrix, with gene expression data suggesting advanced osteogenic differentiation. These results indicate that periosteum-derived cells represent a highly promising cell source for translational bioprinting of bone tissue given their superior osteogenic potential as well as their minimally invasive obtainability.

## 1. Introduction

Segmental bone defects are usually caused by trauma, resection due to benign or malignant tumors, chronic infection, osteonecrosis, or osteodegenerative diseases. They cause severe disabilities in patients [1]. Clinical management of segmental bone defects remains highly challenging, with autologous bone grafts being the gold standard procedures. For smaller defects, autologous free non-vascularized bone grafts or bone graft substitutes can be used, as vascularization is not required. For larger defects, free osteomyocutaneous or myo-osseous free flaps with microsurgical anastomosis can provide vascularized grafts [2,3,4]. The existing methods of reconstructive surgery have many limitations, depending on the underlying pathology and the defect site and size [5]. The rates of perioperative complications, postoperative comorbidities, and functional impairments of donor and recipient site vary from 20 to 50% of all cases, which underlines the need for alternative treatment procedures [6,7].

Advancements in the field of tissue engineering have the potential to offer alternative approaches for the reconstruction of bone defects. Tissue engineering is an interdisciplinary field applying various concepts of life sciences and biotechnological engineering for the manufacture of artificial tissues and organs, offering a diverse toolbox for the construction of personalized human tissues [8]. One of the latest developments is 3D bioprinting, the adaptation of 3D printing for direct fabrication of biological constructs containing living cells. It allows for rapid fabrication of complex structures and manufacturing of more physiological models and customized tissue implants containing multiple cell types and delicate features [9,10,11,12].

For the successful clinical implementation of cell-containing 3D bioprinted bone constructs, selecting the right cell source is essential [13,14,15,16]. The cell source should ideally combine low morbidity of the initial biopsy, an easy harvesting method, rapid expansion, and the ability to differentiate into bone-forming cells [16]. Mesenchymal stem or progenitor cells (MPCs) can be isolated from different body tissues and extensively expanded in vitro while maintaining their undifferentiated, multipotent condition, and have already been investigated in clinical trials [13,17]. MPCs derived from bone marrow and adipose tissue are the ones that have mainly been used for bioprinting artificial bone tissue [13,16,18]. However, the possibility of failure of cell extraction and the inconsistent tissue ossification and speed are barriers to using this procedure in a clinical setting [19,20]. The associated costs and risks may outweigh the long-term benefits.

Periosteum-derived MPCs (P-MPCs) are a promising alternative. They can be obtained via minimally invasive periosteal shaving, and have recently shown high performance in cell-based regenerative therapy of cartilage and bone defects [21]. However, the substantial osteogenic potential of these cells has never before been studied in any bioprinting applications.

We therefore set out to directly compare the osteogenic potential of a panel of human bone-, bone-marrow-, and periosteum-derived mesenchymal progenitor cells, extracted from clinically relevant biopsy sites in a 3D bioprinted environment, and discuss their potential use in a clinical context.

## 2. Results

### 2.1. Bioprinting and Cultivation of Printed Constructs

**Figure 1:**
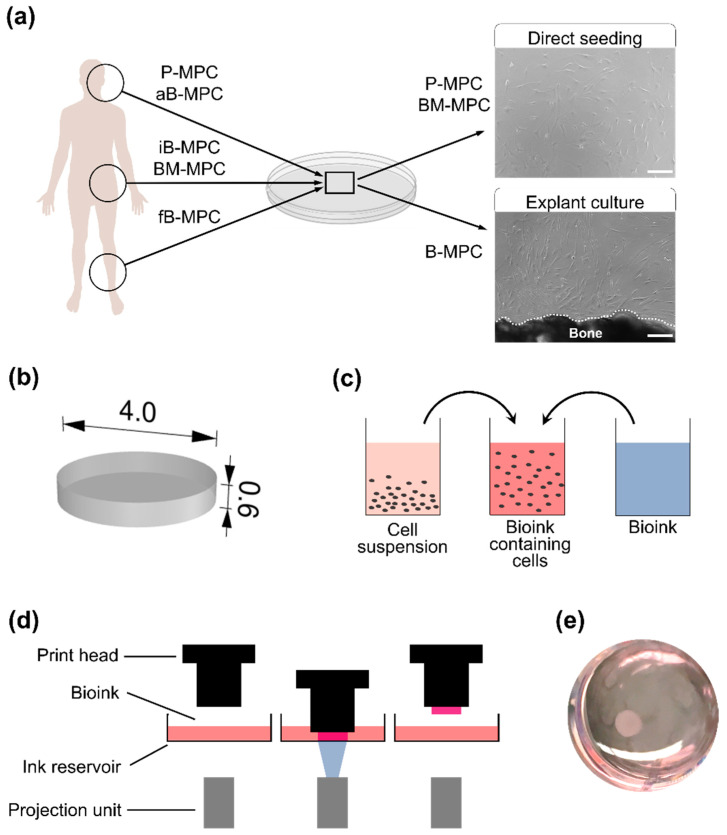
Experimental setup. (**a**) Human mesenchymal progenitor cells were isolated from different body tissues (alveolar bone (aB-MPC), fibula bone (fB-MPC), iliac crest bone (iB-MPC), iliac crest bone marrow (BM-MPC), periosteum of the mastoid (P-MPC)). Scale bar = 200 µm. (**b**) A 3D model was designed with computer-assisted design (CAD) software and exported as an stereolithography (STL) file. Measurements are given in mm. (**c**) Preparation of bioink prior to bioprinting. (**d**) The bioprinting process. The print head is lowered into the ink reservoir and the bioink is solidified by projecting photomasks. (**e**) A photograph showing the completed print in a culture plate.

A panel of human MPC cells was isolated from clinically relevant harvesting sites and expanded (Figure 1a, Table 1). By means of computer-assisted design (CAD) software, a simplified 3D model was designed and exported as a stereolithography (STL) file (Figure 1b). Directly before printing, cells were added to the bioink, which was based on methacrylated gelatin (Figure 1c). Cell-laden constructs were bioprinted using a projection-based stereolithographic printing platform in which precise solidification of hydrogels was achieved by projecting photomasks onto the printing dish, resulting in the fabrication of a three-dimensional construct (Figure 1d,e).

**Figure 2:**
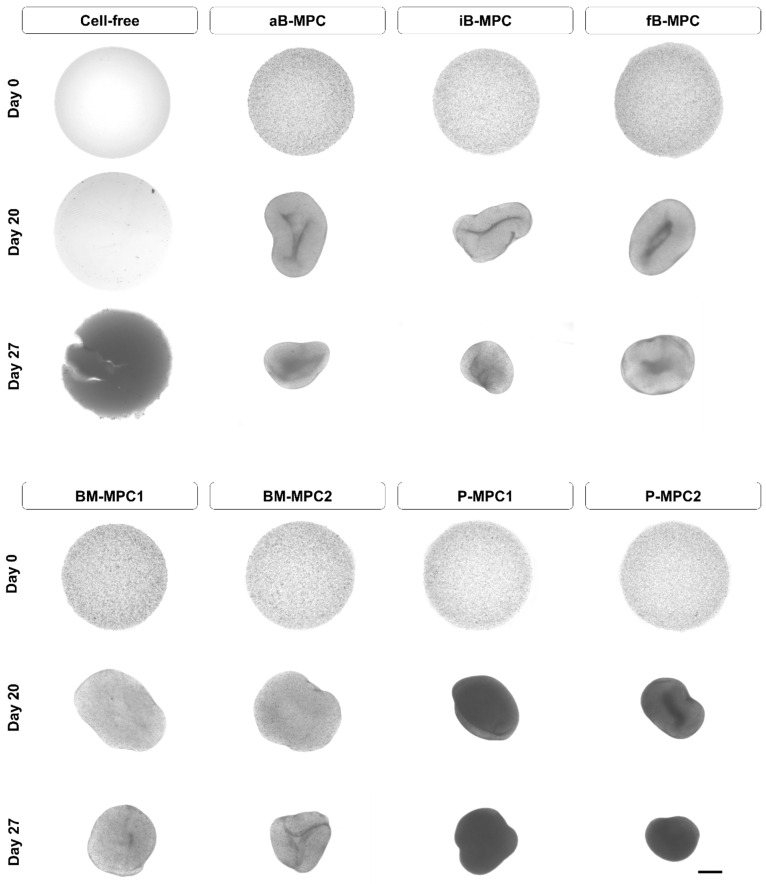
Cultivation of the printed constructs. Microscopic images were taken on days 0, 20, and 27 of cultivation. Scale bar = 1000 µm.

Subsequently, bioprinted constructs were cultivated in osteogenic medium for 28 days. Microscopic images on day 0 showed homogenous distribution in cell-laden constructs (Figure 2). While cell-free bioprints retained their size and discoid shape over the entire cultivation period, shrinkage and central retractions were observed in cell-laden constructs. During cultivation, the P-MPC-laden constructs became progressively less translucent, while the other bioprints retained uniform translucency.

### 2.2. Viability and Metabolic Activity

**Figure 3:**
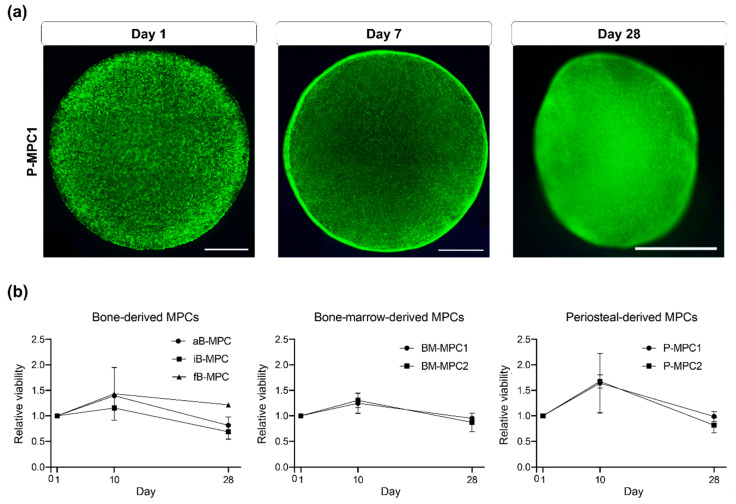
Viability of cells in bioprints. (**a**) Fluorescence microscopy images of propidium iodide/fluorescein diacetate stained constructs showing living cells in green and dead cells in red on days 1, 7, and 28. Representative images shown for P-MPC1. Scale bar = 1000 µm. (**b**) Results of alamarBlue^TM^ assay on days 1, 10, and 28 of cultivation. Viability was normalized to the respective value for each cell type on day 1. *n* = 2.

**Figure A1:**
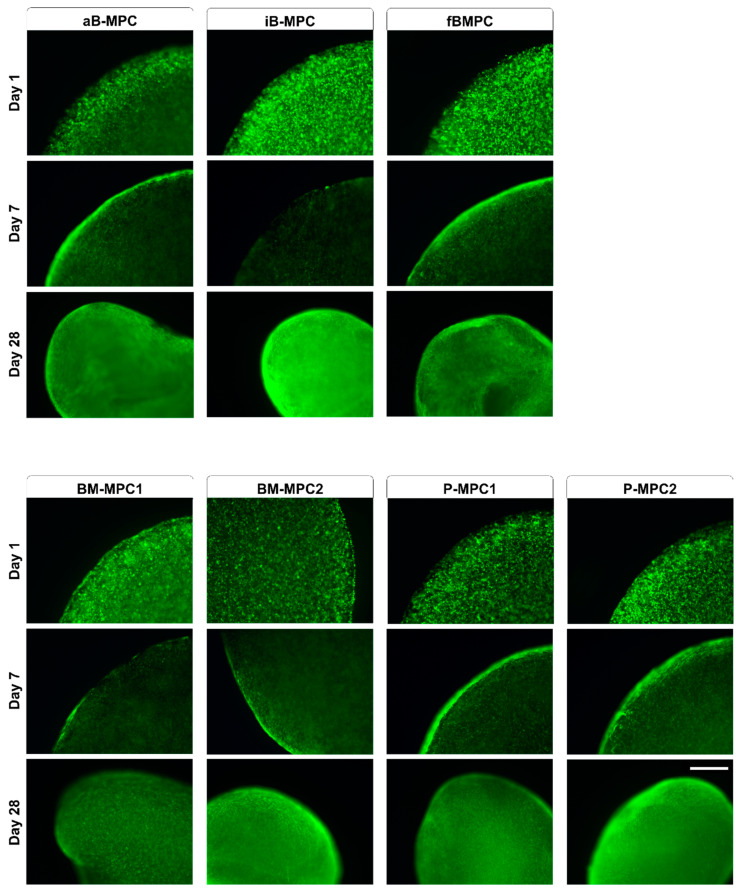
Viability of bioprints. Fluorescence microscopy images of constructs stained with PI/FDA showing living cells in green and dead cells in red on days 1, 7, and 28. Scale bar = 500 µm.

In all cell-laden printed constructs, live/dead staining demonstrated that the vast majority of the cells remained viable throughout the 28 days of culture. Over the entire cultivation period, only a small portion of the cells was found dead (Figure 3a and Figure A1).

A unique feature of bone is its biomechanical properties achieved through the mineralization of the extracellular matrix (ECM). The secretion of ECM depends on the metabolic activity of bone-forming cells. Metabolic activity as a biomarker of viability was assessed using the established alamarBlue^TM^ assay, in which non-fluorescent resazurin is reduced to highly fluorescent resorufin by metabolically active and therefore viable cells [22]. In general, metabolic activity of the bioprinted constructs peaked on day 10 at comparable levels for all subgroups (Figure 3b, Supplementary Material).

### 2.3. Mineralization

Calcification and thus hardening of the bone matrix is accomplished through deposition of mineralized nodules by osteoblasts. This is achieved by incorporating nanoscale calcium phosphate crystals into the osteoid—a soft matrix consisting mainly of collagen I fibrils, previously secreted by the cells [23,24,25].

**Figure 4:**
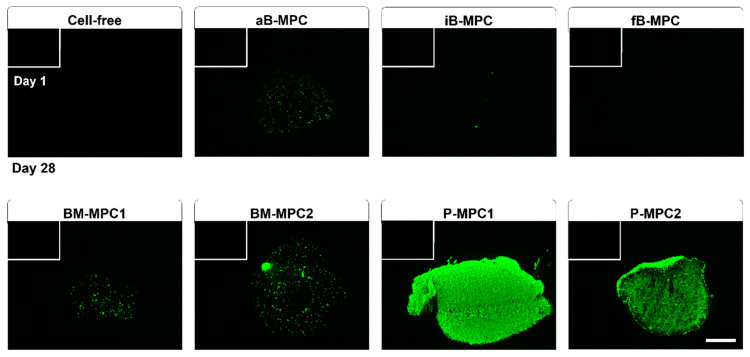
Mineralization of printed constructs. Histological staining on day 1 (white bordered rectangle) and day 28, using OsteoImage™ Mineralization Assay. Scale bar = 500 µm.

Cryosections of the printed constructs were analyzed for deposition of minerals by the cells by staining the hydroxyapatite portion using OsteoImage^TM^ Mineralization Assay (Figure 4). On day 28, the bioprints containing periosteal cells showed a strong uniform signal, while constructs containing BM-MPCs and aB-MPCs displayed formation of nodule-like structures. Only single nodules were visible for iB-MPCs, while no signal was detected for fB-MPCs and for cell-free constructs. On day 1, no signal was detected for any samples.

### 2.4. Gene Expression Analysis

To assess the osteogenic differentiation of printed constructs at the gene expression level, quantitative real-time PCR was performed. Expression of mRNA was normalized to the housekeeping gene TATA-box binding protein (TBP). Alkaline phosphatase (ALPL) is a membrane-bound enzyme involved in bone mineralization through hydrolyzing pyrophosphate, and is a marker for osteogenic differentiation [26,27]. Similarly, collagen I is considered an early bone differentiation marker [28]. It is required for matrix mineralization, as it accounts for most of the organic material in the bone matrix, with collagen type I alpha 1 chain (*COL1A1*) is the predominant collagen [29,30]. *RUNX2* encodes for the Runt-related transcription factor 2, one of the key regulators of osteogenic differentiation, also known as Cbfa1 [28]. The ubiquitously expressed protein osteonectin, encoded by *SPARC* (*s*ecreted protein acidic and cysteine rich), plays a role in the mineralization of the bone matrix and is often used as a late marker of osteogenic differentiation [31]. Osteopontin (*SPP1*; secreted phosphoprotein 1) is one of the SIBLING proteins (small integrin-binding ligand, N-linked glycoproteins) and is also associated with bone mineralization [32].

**Figure 5:**
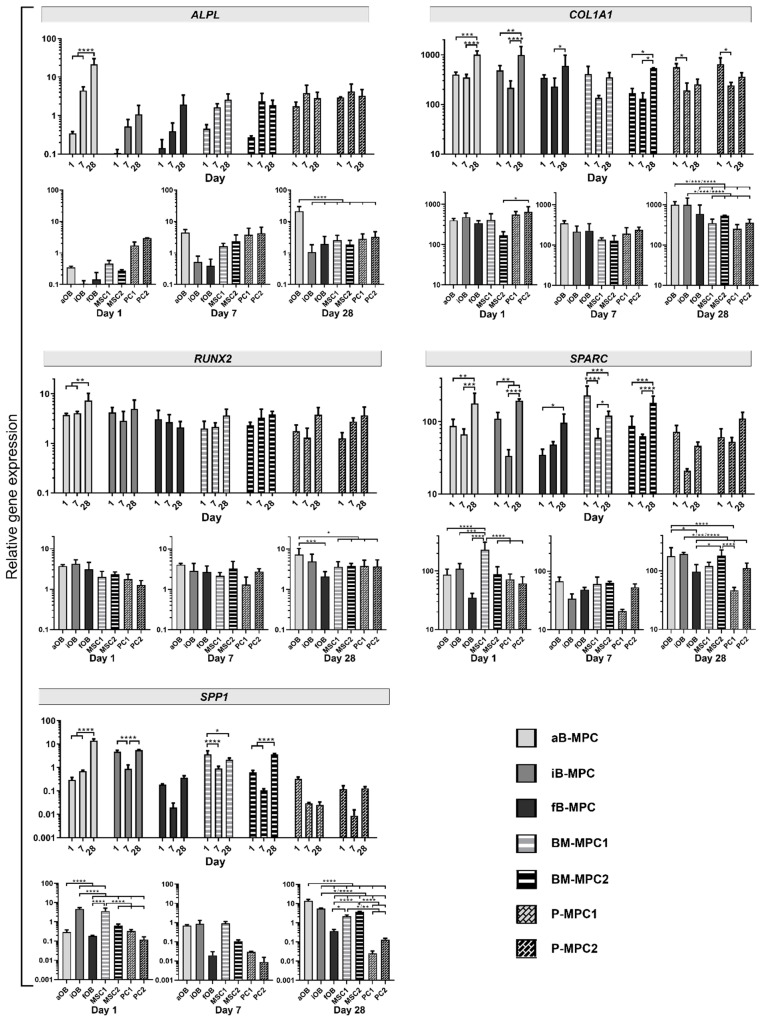
Gene expression analysis. Relative gene expression of differentiation markers *ALPL* (alkaline phosphatase), *COL1A1* (collagen type I alpha chain), *RUNX2* (Runt-related transcription factor 2), *SPARC* (secreted protein acidic and cysteine rich) and *SPP1* (secreted phosphoprotein 1) in bioprinted constructs on days 1, 7, and 28, normalized to TATA-box binding protein (TBP) expression. Asterisks mark statistically significant differences in the data. Data are presented as mean ± standard deviation. *n* = 3.

All the genes of the selected osteogenic marker panel were detected for all cell types. However, expression levels and patterns sometimes differed. Expression of *ALPL* displayed an upward trend over the 28 days of cultivation for all cell types, although this increase was only significant for aB-MPCs (Figure 5, Supplementary Material). A similar increase was observed for expression of *COL1A1* in constructs containing B-MPCs and for BM-MPC donor 2, while BM-MPC donor 1 showed no significant differences. In contrast, bioprints with P-MPCs were found to significantly decrease in *COL1A1* expression from day 1 to day 7 and to remain at a lower level on day 28. Overall, expression levels of both aB-MPCs and iB-MPCs differed significantly from almost all other groups on day 28. *RUNX2* expression levels remained stable over the course of the experiment for all conditions except aB-MPCs, where a significant increase was observed. Constructs containing P-MPCs also exhibited stable expression for *SPARC* and *SPP1*, with lowest expression levels compared to all other conditions on day 28. Donor 1 and 2 samples of BM-MPCs exhibited differences in expression patterns, as for both genes, expression decreased for BM-MPC1, whereas an increase was observed for BM-MPC2. Therefore, BM-MPCs showed a higher donor variability compared to P-MPCs. Expression of *SPARC* increased for all B-MPC bioprints, while for *SPP1*, only aB-MPCs displayed higher gene expression on day 28, with iB-MPCs and fB-MPCs remaining stable overall.

## 3. Discussion

**Figure A2:**
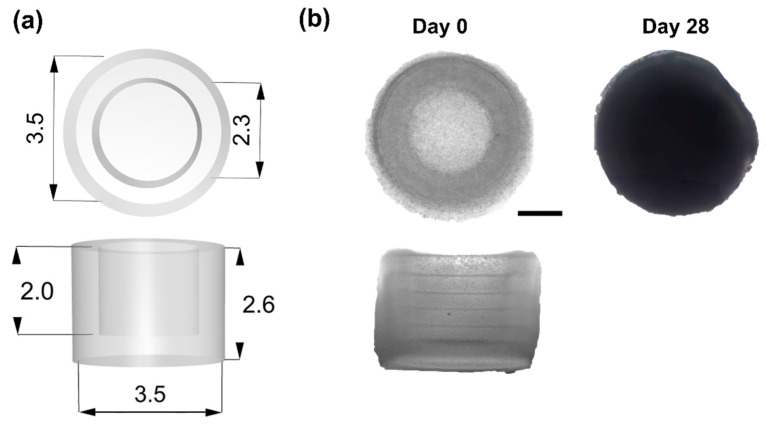
Bioprinting of a scaled-up construct containing P-MPCs. (**a**) CAD model of the bioprinted construct. Measurements are given in mm. (**b**) Microscopic images of bioprints on days 0 and 28 of cultivation. Scale bar = 1000 µm.

In this study, we successfully bioprinted human MPCs isolated from different clinically relevant harvesting sites, and compared their behavior under osteogenic cultivation conditions. For the clinical implementation of cell-containing 3D bioprinted bone constructs, the choice of the right cell source and the adequate bioink are important factors. Methacrylated gelatin (GelMA) based bioink was used to fabricate cell-laden constructs, since it has been shown to be advantageous for cell behavior. As it is an ECM-based material, it naturally presents an arginylglycylaspartic (RGD) amino acid motifs and can be modified by cells [33,34,35]. Here, embedded MPCs were able to contract the gels (Figure 2), implying strong adhesion of the cells to their surrounding matrix. However, similar contraction of the print matrix was not observed for prints with different geometry, suggesting dependence on the overall structure (Figure A2). The multi-layered architecture might improve structural integrity, as we have shown in previous studies [36].

Viability, as measured by alamarBlue^TM^ assay, increased on day 10, followed by a decrease at the end of cultivation, at comparable levels for all cell types (Figure 3). This could be explained by impaired diffusion of the dye through the hydrogel after day 10 (as embedded cells secrete matrix components, including collagen), as well as by the change in construct shape from discoid to spheroid, resulting in a reduced surface area. Changes in measured viability could also result from lowered metabolic activity, as cells lose their highly active proliferative status during differentiation [37]. This is consistent with results from gene expression analysis (Figure 5).

The transcription factor RUNX2 is needed for both differentiation and functioning of osteogenic cells [38,39,40]. Accordingly, gene expression levels remained stable for almost all cell types (Figure 5). A significant increase was observed only for aB-MPCs, hinting at differentiation from early progenitor status to osteoblast-like cells over the course of the experiment. Expression of *COL1A1* is strongly upregulated at the stage prior to matrix mineralization, and its fibril formation is essential for further matrix maturation of bone occurring physiologically in vivo [41]. Hence, bone-derived MPCs appear to be at an early differentiation stage, as an increase of *COL1A1* expression levels was observed over the 28 days of cultivation. These findings are consistent with the OsteoImage^TM^ staining, where little or no mineralization was detected (Figure 4). Accordingly, expression of *ALPL*, one of the early bone differentiation marker genes, was significantly upregulated only in aB-MPCs, while all constructs containing BM-MPCs and P-MPCs showed stable expression levels. In contrast, a downregulation of *COL1A1* expression was observed for P-MPCs. This supports the hypothesis of advanced osteogenic differentiation, since *COL1A1* is usually downregulated during this process [42].

Similarly to collagen, the expression of *SPARC* is upregulated as cells move toward an osteoblast phenotype, and then is subsequently downregulated again [43]. Upregulation of *SPARC* was found for all B-MPC constructs, as well as for BM-MPC2, whereas downregulation was detected for BM-MPC1 and for both P-MPC donors. In contrast, the matrix protein osteopontin, encoded by *SPP1*, is expressed at later stages of osteogenic differentiation and persists at a high level [39]. Again, upregulation was observed for all B-MPC constructs, as well as for BM-MPC2, while expression levels were stable or slightly diminished for P-MPC and BM-MPC1 constructs. Results from gene expression analysis are further substantiated by high mineralization levels for P-MPC constructs, in contrast to moderate mineralization levels for BM-MPC constructs and little to no mineralization for B-MPC constructs, as shown by OsteoImage^TM^ staining (Figure 4).

Directly comparing B-MPCs from different bone entities, cells obtained from the fibula showed the lowest osteogenic potential, since they displayed no mineralization and low expression levels of osteogenic marker genes. Alveolar B-MPCs showed stronger mineralization of the print matrix than those acquired from explant cultures of iliac crest bone. However, donor variability should be considered [44].

To the best of our knowledge, this is the first study that includes periosteum-derived MPCs for the fabrication of artificial bone tissue by 3D bioprinting. Our findings are supported by several other studies, which have shown that periosteal cells are superior in bone and cartilage regeneration compared to bone marrow or other mesenchymal cell sources [45,46,47]. The reported higher proliferation rate of periosteum-derived cells compared to other cell sources is advantageous, as it allows for reduction of the time needed for expansion before transplantation [21]. Furthermore, the periosteum can be removed in a much less invasive way than the other sources of MPCs from various convenient locations, which may facilitate clinical implementation [48,49].

Biocompatible scaffolds secondarily colonized with cartilage or osteoprogenitor cells are already clinically applied in the reconstruction of small cartilage and bone defects [4,50,51]. However, their applicability in large segmental bone defects is limited, as cellular nutrition can only be achieved by diffusion. Implementation of vascularization is currently one of the biggest challenges in the field of tissue engineering. It is crucial for the scale-up of fabricated constructs, and therefore for their potential for transplantation, since diffusion limits need to be overcome to ensure supply with nutrients and oxygen and removal of waste products [52,53,54]. Recently, Thomas et al. presented an enzyme-based system for rapid fabrication of vasculature-like structures for multi-material stereolithographic bioprinters [55]. Perfusable channels can be printed in a bulk material using hyaluronic acid-based ink, which is first solidified and then digested using hyaluronidase. These channels can optionally be lined with endothelial cells by embedding the cells in the channel ink and subsequently releasing them via enzymatic digestion of the surrounding matrix, allowing cell attachment to the resulting channel walls. This system could readily be adapted to our application, allowing bigger constructs to be fabricated. Furthermore, the introduction of endothelial cells can have a positive effect on the osteogenic maturation of tissues, as has been shown before [36,56,57].

Further differentiation of printed cells might be observed by prolonging the cultivation time. This would be particularly interesting for bone-derived cells, as gene expression and mineralization data indicate an early differentiation stage. Moreover, loading the hydrogel with cell attractants like growth factors or chemokines could enhance the in vitro maturation of the bioprinted constructs and be advantageous for subsequent transplantation, as has been shown before [58,59,60,61,62]. This should be validated in follow-up in vivo studies.

For successful translation, several challenges need to be met. Autologous transplants require a large number of cells, necessitating sophisticated cell isolation and expansion workflows. Furthermore, the material and its printed structure need to promote engraftment after implantation. For GelMA, the material used in this study, previous publications have reported promising results [62,63]. Another aspect to consider is the ease of handling of the fabricated constructs in the surgical procedure. Prior to clinical application, long-term in vitro testing, as well as in vivo studies, must be completed, proving biocompatibility for prolonged time periods.

Although much larger artificial bone grafts are required to restore segmental bone defects, the purpose of this study was to compare the osteogenic potential of MPCs from different bone entities in 3D bioprinted constructs first. Therefore, a simplified design was chosen, allowing for screening of MPCs originating from four bone entities (alveolar, fibula, iliac crest, and mastoid) using three different extraction and harvesting methods (explant culture, bone marrow aspiration, and periosteal shaving with subsequent seeding). To facilitate investigation of a large number of relevant bone sites, a total of only seven donors were analyzed. For better significance, the number of donors per bone entity should be increased. However, homogenous results were still observed from MPCs in the different entities when exposed to the same fabrication and cultivation protocols within our study.

In addition to the origin of the osteogenic cell source, the biomechanical characteristics of different implant types should also be thoroughly assessed in the future. However, this was beyond the scope of this study, since these properties are highly dependent on the construct size, and more sensible data could be obtained by bioprinting structures resembling the prospective implant features more closely.

## 4. Materials and Methods

### 4.1. Photoink Synthesis

Methacrylated gelatin (GelMA) was synthesized as described previously [64,65]. After dissolution at 10% *w/v* in phosphate-buffered saline (PBS), type A gelatin from porcine skin (300 bloom) was heated to 50 °C, and methacrylic anhydride was added dropwise at 0.1 mL g^−1^ gelatin. The reaction was allowed to continue for three hours under constant stirring. After adjusting the pH to 7.4, dialysis of GelMA was performed for four days against distilled water through a 12–14 kDa cut-off membrane to remove the remaining methacrylate and salts. After lyophilization at 1 mbar and −60 °C, GelMA was stored at −20 °C. Methacrylation was measured by 1H–NMR using a Bruker Avance III at 500 MHz (Bruker Corporation, Billerica, MA, USA). Lithium phenyl-2,4,6-trimethylbenzoyl phosphinate (LAP) was used as a photoinitiator. Synthesis was performed as previously published [66,67]. All synthesis reagents were purchased from Sigma-Aldrich (Saint Louis, MO, USA).

### 4.2. Cell Isolation and Culture

#### 4.2.1. Ethical Statement

All subjects gave their informed consent for inclusion before they participated in the study. The study was conducted in accordance with the Declaration of Helsinki, and the protocol was approved by the Ethics Committee of Charité-Universitätsmedizin Berlin (EA4/049/13, EA2/068/14).

#### 4.2.2. Bone Marrow MPCs

Adult human BM-MPCs were isolated from iliac crest bone marrow aspirates according to a protocol published previously [68]. Heparinized aspirates were diluted in BM-MPC medium and seeded directly in tissue culture flasks (1 mL undiluted aspirate per 175 cm^2^). BM-MPC medium was composed of Dulbecco’s modified Eagle’s medium (DMEM) containing 1 g L^−1^ glucose, 2 mM L-alanyl-L-glutamine, 100 U mL^−1^ penicillin, 100 µg mL^−1^ streptomycin, 20 mM HEPES (all Merck, Berlin, Germany), 10% fetal calf serum (FCS, HyClone^TM^, GE Healthcare, Chicago, IL, USA), and 2 ng mL^−1^ basic fibroblast growth factor (FGF2, PeproTech, Rockyhill, CT, USA). Adherent cells were propagated in medium that was exchanged three times a week; non-adherent mononuclear cells were removed by media exchange. Cells were maintained at 37 °C in a humidified atmosphere with 5% CO_2_, and were trypsinized upon reaching a confluence of 90% by means of a 0.05% trypsin-EDTA solution (Merck, Berlin, Germany).

#### 4.2.3. Bone-Derived MPCs

Adult primary human bone-derived MPCs were isolated from cancellous bone of patients undergoing free vascularized fibula tissue transfer, free vascularized iliac crest tissue transfer, or dental implantation, following slightly modified protocols published previously [44,69]. The bone tissue was repeatedly washed with PBS to remove blood components under a sterile workbench. Subsequently, the explants were minced and seeded in tissue culture flasks containing cell culture medium. The cell culture medium was composed of DMEM containing 1 g L^−1^ glucose, 2 mM L-alanyl-L-glutamine, 100 U mL^−1^ penicillin, 10 µg mL^−1^ streptomycin, 20 mM HEPES (all Merck, Berlin, Germany), 10% FCS (HycloneTM, GE Healthcare, Chicago, IL, USA), and 2 ng mL^−1^ basic fibroblast growth factor (FGF2, PeproTech, Rockyhill, CT, USA). Explants were cultured in a humidified incubator at 37 °C with 5% CO_2_. Cells started to grow out within 3–7 days and reached critical confluency on or after day 14. Non-adherent cells were removed by media exchange, which was conducted three times a week. At confluency, the culture was expanded by trypsinization by means of 0.05% trypsin-EDTA solution (Merck, Berlin, Germany).

#### 4.2.4. Periosteum-Derived MPCs

Periosteal tissues (0.5 cm^2^) were harvested according to a method previously described [46] from the human mastoid of two patients undergoing mastoidectomy. In brief, the periosteal flap was rinsed with Hank’s solution (Merck, Berlin, Germany) three times, minced and digested for 3 h in DMEM/Ham’s F12 medium (Merck, Berlin, Germany) containing 10,000 U mL^−1^ collagenase II (Merck, Berlin, Germany), 10% human allogenic serum (German Red Cross, Berlin, Germany), 2.5% HEPES (Biochrom, Berlin, Germany), 100 U mL^−1^ penicillin, and 10 µg mL^−1^ streptomycin (Biochrom, Berlin, Germany). Subsequently, the cells were harvested, resuspended in DMEM/Ham’s F12 medium containing 10% human allogenic serum, plated in cell culture flasks, and allowed to attach for about 4–6 days. Non-adherent cells were removed by exchange of medium. Adherent growing periosteum-derived MPCs (P-MPCs) were sub-cultured under standard cell culture conditions. At 90% confluence, P-MPCs were detached by treatment with 0.05% trypsin-EDTA, replated, and sub-cultured in DMEM medium containing 10% FCS.

**Figure A3:**
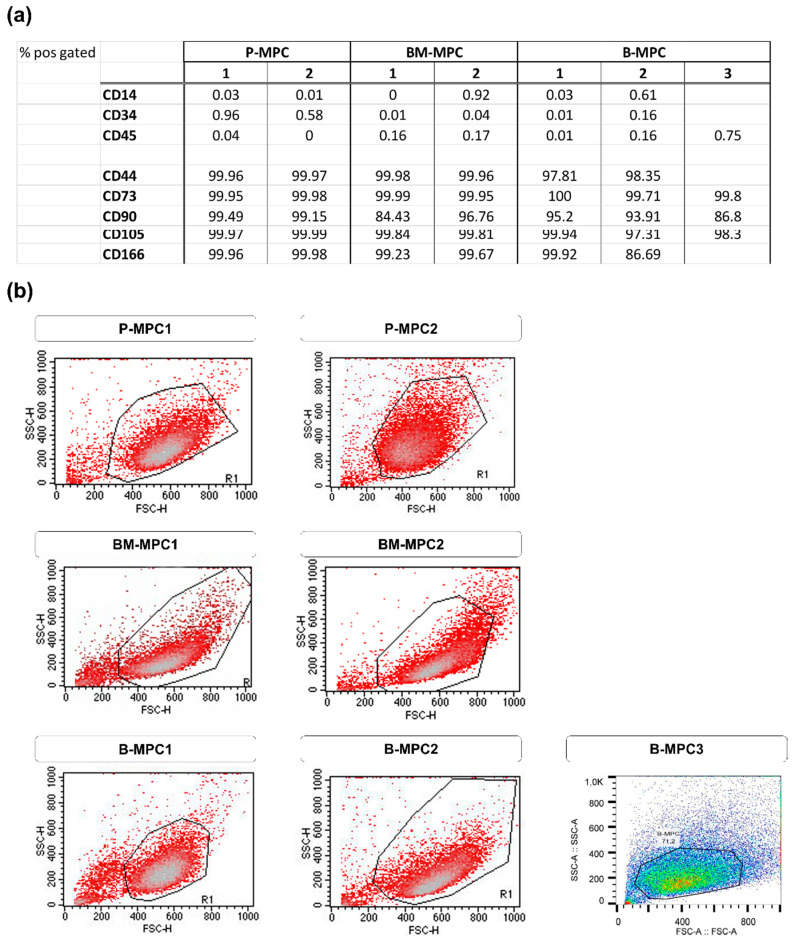
Flowcytometrical analyses of MPCs. (**a**) Results from flowcytometrical analyses of cell surface antigen patterns of isolated MPCs. (**b**) Side versus forward scatter density plots of analyzed MPCs.

A summary of the cells types used and their sources are given in Table 1. Cells were characterized by flowcytometrical analyses of cell surface antigens as described previously (Figure A3) [70]. All cells were used at passage 3. Consumables were obtained from Corning Inc. (Corning, CA, USA) unless stated otherwise.

### 4.3. Bioprinting

3D models were designed using Rhinoceros 6 software (Robert McNeel and Associates, Seattle, WA, USA) and exported as an STL file. Photomasks were generated using the printer’s software. Photoink was prepared by dissolving lyophilized GelMA in PBS and then diluting it to the final concentration of 8% *w/w* while adding 0.1% *w/w* LAP. To fabricate cell-laden constructs, cells were detached from the culture dish and added to the ink at 20 × 10^6^ cells mL^−1^ directly before printing. Bioprinting was performed using a proprietary stereolithographic printing platform, as described previously [10,55]. Briefly, precise solidification of hydrogels was achieved by projecting photomasks onto the printing dish, resulting in the fabrication of a three-dimensional construct.

### 4.4. Bioprint Cultivation

The printed constructs were cultivated for 28 days in osteogenic medium (DMEM with 1 g L^−1^ glucose, 10% FCS, 2.5% HEPES, 100 U mL^−1^ penicillin, 10 µg mL^−1^ streptomycin, 100 nM dexamethasone (Sigma-Aldrich, Saint Louis, MO, USA), 0.05 mM L-ascorbic acid 2-phosphate (Sigma-Aldrich, Saint Louis, MO, USA), and 10 mM β-glycerophosphate (Sigma-Aldrich, Saint Louis, MO, USA)) in 24-well ultra-low attachment multiple well plates. Medium exchange was performed three times a week. Images were taken using the BIOREVO BZ-9000 microscope (Keyence, Osaka, Japan) and CK40 (Olympus, Hamburg, Germany).

### 4.5. Assessment of Viability

#### 4.5.1. alamarBlue^TM^ Assay

To evaluate cell viability after printing, alamarBlue^TM^ assay was used according to the manufacturer’s recommendations (Thermo Fisher Scientific, Waltham, MA, USA). AlamarBlue^TM^ was diluted 1:10 in DMEM containing 10% FCS (AB medium). The medium was removed and each construct was incubated in 500 μL AB medium for 4 h at 37 °C and 5% CO_2_. After the incubation period, 4 × 100 μL of each well was transferred to a 96-well plate. Fluorescence was measured in a plate reader with the following wavelength filter settings—540 nm for excitation and 590 nm for emission. AB medium without any cells served as a blank measurement. Two bioprinted constructs per condition were analyzed and measured in technical quadruplicates.

#### 4.5.2. Live/Dead Staining

Whole constructs were examined for viability using propidium iodide/fluorescein diacetate staining (PI/FDA; Sigma-Aldrich, Saint Louis, MO, USA) on days 1, 7, and 28 of osteogenic maintenance. After washing with PBS (Merck, Berlin, Germany), staining was performed, first in an FDA solution (3 μg mL^−1^; 15 min, 37 °C) and then in a PI staining solution (100 μg mL^−1^; 2 min; room temperature). For microscopy, an Olympus CKX41 combined with a reflected fluorescence microscopy system was used (Olympus, Hamburg, Germany). The staining results were photodocumented using the ProgRes^®^ speed XT core 5 camera and ProgRes^®^ CapturePro 2.10 software (both Jenoptik, Jena, Germany).

### 4.6. Mineralization

The printed constructs were washed in PBS and fixated for 15 min at room temperature using 4% formalin (ROTI^®^Histofix; Roth, Karlsruhe, Germany). After washing in PBS, the printed constructs were embedded in an optimal cutting temperature (OCT) compound (Sakura Finetek, Alphen aan den Rijn, Netherlands) and incubated at 37 °C for 35 min. Samples were shock-frozen in liquid nitrogen and stored at −80 °C until further use. Following this, 10 µm cryosections were produced using the CM1950 cryostat (Leica Microsystems, Wetzlar, Germany). To assess the mineralization of the printed matrix, samples were stained using the OsteoImage^TM^ Mineralization Assay (Lonza, Basel, Switzerland) according to the manufacturer’s protocol. Briefly, sections were permeabilized with acetone for 10 min at −20 °C, washed two times with PBS and once with a wash buffer, incubated with the staining reagent for 30 min at room temperature, washed three times with a wash buffer, and mounted using ImsolMount (ImmunoLogic, Duiven, Netherlands). Images were taken using the BIOREVO BZ-9000 microscope (Keyence, Osaka, Japan).

### 4.7. Real-Time PCR

To assess the relative gene expression levels of bone-specific marker genes, a semi-quantitative real-time PCR was performed. Three bioprints per condition were pooled, isolated, and analyzed. Isolation of total mRNA was performed using the ARCTURUS^®^ PicoPure^TM^ RNA isolation kit, following the manufacturer’s protocol (Thermo Fisher Scientific, Waltham, MA, USA). mRNA was quantified using NanoDrop^®^ ND-1000 spectrophotometer, and transcribed to cDNA using the iScript^TM^ cDNA synthesis kit (Bio-Rad, Munich, Germany) according to the manufacturer’s protocol. Real-time PCR was performed using the CFX96 real-time PCR system (Bio-Rad, Munich, Germany). Primer (10 µM), cDNA (equivalent to 6 ng total mRNA), and SensiFAST^TM^ SYBR^®^ No-ROX qPCR master mix (Bioline, Luckenwalde, Germany) were mixed in a total volume of 20 µL. After each PCR run, a melting curve analysis was performed to exclude non-specific amplification. Three replicates of each sample were measured. The relative expression of marker genes was normalized to the housekeeping gene TATA-box binding protein (*TBP*). The primer sequences are given in Table 2.

### 4.8. Statistical Analysis

Statistical analyses were performed using GraphPad Prism 8 (San Diego, CA, USA). All values are given as mean ± standard deviation. AlamarBlue^TM^ assay and real-time PCR data were analyzed using two-way ANOVA with Tukey’s multiple comparison test (Supplementary Material). P values smaller than or equal to 0.05 were considered significant.

## 5. Conclusions

In our work we compared osteogenic cells from different bone entities in terms of their applicability in biofabrication of autologous bone implants. Our findings suggest that periosteum-derived MPCs are the most suitable cell source for 3D bioprinted bone constructs based on microscopic observations, viability, mineralization capacity, and gene expression analysis, indicating an advanced differentiation stage with strong mineralization of the surrounding matrix. Additionally, these cells are readily obtainable via minimally invasive periosteal shaving and show high proliferation rates, making them ideal candidates for translational applications. The unique combination of these advantages makes the use of periosteum-derived MPCs a promising approach for the fabrication of autologous 3D bioprinted bone grafts.

## Figures and Tables

**Table 1:** Cell types used for bioprinting.

Construct	Cell Type	Biopsy Site	Biopsy Method	Expansion Method
Cell-free	–	–	–	–
aB-MPC	Bone-derived MPC	Alveolar bone	Bone explantation	Explant outgrowth
iB-MPC		Iliac crest		
fB-MPC		Fibula		
BM-MPC1	Bone marrow MPC	Iliac crest bone marrow	Fine needle aspiration	Direct seeding
BM-MPC2				
P-MPC1	Periosteal MPC	Mastoid	Periosteum explantation	Seeding after tissue digestion
P-MPC2				

**Table 2:** Sequences of primers used for real-time PCR.

Gene	Accession Number	Description	For	Rev
*ALPL*	NM_000478	Alkaline phosphatase	cccacttcatctggaaccgc	ccgtggtcaattctgcctcc
*COL1A1*	NM_000088	Collagen type I alpha 1 chain	gccgtgacctcaagatgtg	gccgaaccagacatgcctc
*RUNX2*	NM_001015051	Runt-related transcription factor 2	tcacaaatcctccccaagtagc	ggcgggacacctactctcatac
*SPARC*	NM_003118	Secreted protein acidic and cysteine rich	gcagaagctgcgggtgaagaa	ctcgaaaaagcgggtggtgc
*SPP1*	NM_000582	Secreted phosphoprotein 1	cactgattttcccacggacct	ccattcaactcctcgctttcc
*TBP*	NM_003194	TATA-box binding protein	ccttgtgctcacccaccaac	tcgtcttcctgaatccctttagaatag

## Data Availability

The data presented in this study are available in the supplementary material. PCR data is available from the corresponding author upon request.

## Supplementary Materials

The following are available online at https://www.mdpi.com/1422-0067/22/2/796/s1. The data presented in this study are available from the corresponding author upon request.

## Abbreviations

3D	Three-dimensional
1H-NMR	Proton nuclear magnetic resonance
a	Alveolar bone
AB	alamarBlue^TM^
B-MPC	Bone-derived mesenchymal progenitor cell
BM-MPC	Bone-marrow-derived mesenchymal progenitor cell
CAD	Computer-aided design
CO_2_	Carbon dioxide
DMEM	Dulbecco’s modified Eagle’s medium
ECM	Extracellular matrix
EDTA	Ethylenediaminetetraacetic acid
f	Fibular bone
FCS	Fetal calf serum
FGF	Fibroblast growth factor
GelMA	Methacrylated gelatin
HEPES	Hydroxyethyl piperazineethanesulfonic acid
i	Iliac-crest bone
kDa	Kilodalton
LAP	Lithium phenyl-2,4,6-trimethylbenzoylphosphinate
min	Minute
mL	Milliliter
MHz	Megahertz
MPC	Mesenchymal progenitor cell
mRNA	Messenger ribonucleic acid
µL	Microliter
nm	Nanometer
PBS	Phosphate-buffered saline
PI/FDA	Propidium iodide/fluorescein diacetate
P-MPC	Periosteum-derived mesenchymal progenitor cell
RGD	Tripeptide Arg-Gly-Asp
STL	Stereolithography
