# Supplementary material for: Comparison of the Translational Potential of Human Mesenchymal Progenitor Cells from Different Bone Entities for Autologous 3D Bioprinted Bone Grafts
Source: Int J Mol Sci. 2021 Jan 14;22(2):796. doi: 10.3390/ijms22020796 (PMC7830021; doi:10.3390/ijms22020796)
Supplement: Supplementary file 1 [file ijms-22-00796-s001.zip › P values alamarBlue.pdf]

**alamarBlue**	aB-MPC	iB-MPC	fB-MPC	BM-MPC1	BM-MPC2	P-MPC1	P-MPC2
d1 vs. d10	0,1313	0,7158	0,0915	0,4288	0,2877	**0,0095**	**0,0063**
d1 vs. d28	0,61	0,2669	0,5197	0,9652	0,7936	0,9984	0,6264
d10 vs. d28	**0,0187**	0,0655	0,5196	0,3019	0,0937	**0,0084**	**0,0007**

Day 1	aB-MPC	iB-MPC	fB-MPC	BM-MPC1	BM-MPC2	P-MPC1	P-MPC2
aB-MPC	-						
iB-MPC	>0,9999	-					
fB-MPC	>0,9999	>0,9999	-				
BM-MPC1	>0,9999	>0,9999	>0,9999	-			
BM-MPC2	>0,9999	>0,9999	>0,9999	>0,9999	-		
P-MPC1	>0,9999	>0,9999	>0,9999	>0,9999	>0,9999	-	
P-MPC2	>0,9999	>0,9999	>0,9999	>0,9999	>0,9999	>0,9999	-

Day 10	aB-MPC	iB-MPC	fB-MPC	BM-MPC1	BM-MPC2	P-MPC1	P-MPC2
aB-MPC	-						
iB-MPC	0,8714	-					
fB-MPC	>0,9999	0,7796	-				
BM-MPC1	0,9867	0,9989	0,9593	-			
BM-MPC2	0,999	0,9858	0,9933	>0,9999	-		
P-MPC1	0,8618	0,2097	0,9309	0,4336	0,6043	-	
P-MPC2	0,7733	0,1523	0,8665	0,3368	0,4937	>0,9999	-

Day 28	aB-MPC	iB-MPC	fB-MPC	BM-MPC1	BM-MPC2	P-MPC1	P-MPC2
aB-MPC	-						
iB-MPC	0,9944	-					
fB-MPC	0,4056	0,1444	-				
BM-MPC1	0,9908	0,8218	0,815	-			
BM-MPC2	>0,9999	0,9593	0,5868	0,9996	-		
P-MPC1	0,9677	0,7146	0,9003	>0,9999	0,9962	-	
P-MPC2	>0,9999	0,993	0,4204	0,9925	>0,9999	0,9721	-
