# Supplementary material for: Comparison of the Translational Potential of Human Mesenchymal Progenitor Cells from Different Bone Entities for Autologous 3D Bioprinted Bone Grafts
Source: Int J Mol Sci. 2021 Jan 14;22(2):796. doi: 10.3390/ijms22020796 (PMC7830021; doi:10.3390/ijms22020796)
Supplement: Supplementary file 1 [file ijms-22-00796-s001.zip › P values real-time PCR.pdf]

ALPL	aB-MPC	iB-MPC	fB-MPC	BM-MPC1	BM-MPC2	P-MPC1	P-MPC2
d0 vs. d7	0,0623	0,9702	0,9892	0,784	0,4685	0,4727	0,7594
d0 vs. d28	**<0,0001**	0,8446	0,5702	0,4539	0,64	0,8097	0,9822
d7 vs. d28	**<0,0001**	0,945	0,6577	0,8503	0,9578	0,8433	0,8578

ALPL day 1	aB-MPC	iB-MPC	fB-MPC	BM-MPC1	BM-MPC2	P-MPC1	P-MPC2
aB-MPC	-						
iB-MPC	>0,9999	-					
fB-MPC	>0,9999	>0,9999	-				
BM-MPC1	>0,9999	>0,9999	>0,9999	-			
BM-MPC2	>0,9999	>0,9999	>0,9999	>0,9999	-		
P-MPC1	0,9842	0,9656	0,9693	0,9898	0,9798	-	
P-MPC2	0,75	0,6689	0,6823	0,7859	0,7269	0,9923	-

ALPL day 7	aB-MPC	iB-MPC	fB-MPC	BM-MPC1	BM-MPC2	P-MPC1	P-MPC2
aB-MPC	-						
iB-MPC	0,3012	-					
fB-MPC	0,2662	>0,9999	-				
BM-MPC1	0,6826	0,9954	0,9917	-			
BM-MPC2	0,8956	0,94	0,919	0,9996	-		
P-MPC1	0,9998	0,5086	0,4631	0,8734	0,9806	-	
P-MPC2	>0,9999	0,3727	0,333	0,7623	0,9383	>0,9999	-

ALPL day 28	aB-MPC	iB-MPC	fB-MPC	BM-MPC1	BM-MPC2	P-MPC1	P-MPC2
aB-MPC	-						
iB-MPC	**<0,0001**	-					
fB-MPC	**<0,0001**	0,9989	-				
BM-MPC1	**<0,0001**	0,9773	0,9998	-			
BM-MPC2	**<0,0001**	0,9993	>0,9999	0,9996	-		
P-MPC1	**<0,0001**	0,9522	0,9986	>0,9999	0,9978	-	
P-MPC2	**<0,0001**	0,8708	0,9871	0,9997	0,9834	>0,9999	-

COL1A1	aB-MPC	iB-MPC	fB-MPC	BM-MPC1	BM-MPC2	P-MPC1	P-MPC2
d0 vs. d7	0,9185	0,128	0,6796	0,115	0,953	**0,0242**	**0,0113**
d0 vs. d28	**0,0002**	**0,0013**	0,1589	0,8929	**0,0239**	0,0701	0,0901
d7 vs. d28	**<0,0001**	**<0,0001**	**0,0254**	0,2625	**0,0112**	0,8916	0,6605

COL1A1 day 1	aB-MPC	iB-MPC	fB-MPC	BM-MPC1	BM-MPC2	P-MPC1	P-MPC2
aB-MPC	-						
iB-MPC	0,9962	-					
fB-MPC	0,9994	0,9407	-				
BM-MPC1	>0,9999	0,9982	0,9986	-			
BM-MPC2	0,6127	0,2585	0,8581	0,5635	-		
P-MPC1	0,8946	0,9971	0,6695	0,9209	0,0795	-	
P-MPC2	0,5234	0,8741	0,2759	0,5725	**0,015**	0,994	-

COL1A1 day 7	aB-MPC	iB-MPC	fB-MPC	BM-MPC1	BM-MPC2	P-MPC1	P-MPC2
aB-MPC	-						
iB-MPC	0,9547	-					
fB-MPC	0,9734	>0,9999	-				
BM-MPC1	0,7007	0,9969	0,9925	-			
BM-MPC2	0,6746	0,9954	0,9897	>0,9999	-		
P-MPC1	0,9072	>0,9999	>0,9999	0,9995	0,9992	-	
P-MPC2	0,9848	>0,9999	>0,9999	0,9855	0,981	0,9998	-

COL1A1 day 28	aB-MPC	iB-MPC	fB-MPC	BM-MPC1	BM-MPC2	P-MPC1	P-MPC2
aB-MPC	-						
iB-MPC	>0,9999	-					
fB-MPC	0,0592	0,067	-				
BM-MPC1	**0,0003**	**0,0004**	0,5438	-			
BM-MPC2	**0,021**	**0,0241**	0,9996	0,7953	-		
P-MPC1	**<0,0001**	**<0,0001**	0,1748	0,9911	0,361	-	
P-MPC2	**0,0004**	**0,0005**	0,5874	>0,9999	0,8297	0,9857	-

RUNX2	aB-MPC	iB-MPC	fB-MPC	BM-MPC1	BM-MPC2	P-MPC1	P-MPC2
d0 vs. d7	0,9362	0,4137	0,9103	0,9902	0,6285	0,8971	0,3463
d0 vs. d28	**0,0037**	0,758	0,6042	0,2777	0,3323	0,1395	0,0681
d7 vs. d28	**0,0095**	0,1266	0,8474	0,3409	0,8661	0,0551	0,6535

RUNX2 day 1	aB-MPC	iB-MPC	fB-MPC	BM-MPC1	BM-MPC2	P-MPC1	P-MPC2
aB-MPC	-						
iB-MPC	0,9992	-					
fB-MPC	0,9967	0,9388	-				
BM-MPC1	0,6642	0,3796	0,9426	-			
BM-MPC2	0,8355	0,5667	0,9897	>0,9999	-		
P-MPC1	0,5149	0,2594	0,8621	>0,9999	0,9981	-	
P-MPC2	0,2467	0,0988	0,586	0,9908	0,9464	0,9989	-

RUNX2 day 7	aB-MPC	iB-MPC	fB-MPC	BM-MPC1	BM-MPC2	P-MPC1	P-MPC2
aB-MPC	-						
iB-MPC	0,9008	-					
fB-MPC	0,8223	>0,9999	-				
BM-MPC1	0,528	0,9935	0,9989	-			
BM-MPC2	0,9884	0,9995	0,9963	0,9266	-		
P-MPC1	0,136	0,7534	0,8481	0,9825	0,4888	-	
P-MPC2	0,8545	>0,9999	>0,9999	0,9977	0,9981	0,8152	-

RUNX2 day 28	aB-MPC	iB-MPC	fB-MPC	BM-MPC1	BM-MPC2	P-MPC1	P-MPC2
aB-MPC	-						
iB-MPC	0,2782	-					
fB-MPC	**0,0002**	0,1172	-				
BM-MPC1	**0,0166**	0,872	0,7555	-			
BM-MPC2	**0,0272**	0,9365	0,6418	>0,9999	-		
P-MPC1	**0,0252**	0,9282	0,6604	>0,9999	>0,9999	-	
P-MPC2	**0,0177**	0,8811	0,7426	>0,9999	>0,9999	>0,9999	-

SPARC	aB-MPC	iB-MPC	fB-MPC	BM-MPC1	BM-MPC2	P-MPC1	P-MPC2
d0 vs. d7	0,6862	**0,0095**	0,8477	**<0,0001**	0,5766	0,1016	0,9393
d0 vs. d28	**0,0017**	**0,0032**	**0,0408**	**0,0002**	**0,001**	0,5508	0,1191
d7 vs. d28	**0,0001**	**<0,0001**	0,1326	**0,0443**	**<0,0001**	0,548	0,0584

SPARC day 1	aB-MPC	iB-MPC	fB-MPC	BM-MPC1	BM-MPC2	P-MPC1	P-MPC2
aB-MPC	-						
iB-MPC	0,9675	-					
fB-MPC	0,3544	0,056	-				
BM-MPC1	**<0,0001**	**0,0002**	**<0,0001**	-			
BM-MPC2	>0,9999	0,9723	0,3385	**<0,0001**	-		
P-MPC1	0,9964	0,7318	0,726	**<0,0001**	0,9954	-	
P-MPC2	0,9365	0,4438	0,9329	**<0,0001**	0,9282	0,9992	-

SPARC day 7	aB-MPC	iB-MPC	fB-MPC	BM-MPC1	BM-MPC2	P-MPC1	P-MPC2
aB-MPC	-						
iB-MPC	0,8248	-					
fB-MPC	0,9883	0,9965	-				
BM-MPC1	>0,9999	0,9297	0,9989	-			
BM-MPC2	>0,9999	0,8895	0,9963	>0,9999	-		
P-MPC1	0,509	0,9983	0,9179	0,679	0,6035	-	
P-MPC2	0,9975	0,9853	>0,9999	>0,9999	0,9995	0,8447	-

SPARC day 28	aB-MPC	iB-MPC	fB-MPC	BM-MPC1	BM-MPC2	P-MPC1	P-MPC2
aB-MPC	-						
iB-MPC	0,9922	-					
fB-MPC	**0,0278**	**0,0041**	-				
BM-MPC1	0,2536	0,059	0,9516	-			
BM-MPC2	>0,9999	0,999	**0,0158**	0,17	-		
P-MPC1	**<0,0001**	**<0,0001**	0,4078	0,0578	**<0,0001**	-	
P-MPC2	0,1103	**0,0204**	0,9972	0,9995	0,0682	0,1476	-

SPP1	aB-MPC	iB-MPC	fB-MPC	BM-MPC1	BM-MPC2	P-MPC1	P-MPC2
d0 vs. d7	0,7539	**<0,0001**	0,9509	**<0,0001**	0,6226	0,8522	0,9783
d0 vs. d28	**<0,0001**	0,2914	0,947	**0,0246**	**<0,0001**	0,8488	0,9999
d7 vs. d28	**<0,0001**	**<0,0001**	0,8117	0,0773	**<0,0001**	>0,9999	0,975

SPP1 day 1	aB-MPC	iB-MPC	fB-MPC	BM-MPC1	BM-MPC2	P-MPC1	P-MPC2
aB-MPC	-						
iB-MPC	**<0,0001**	-					
fB-MPC	>0,9999	**<0,0001**	-				
BM-MPC1	**<0,0001**	0,5961	**<0,0001**	-			
BM-MPC2	0,9965	**<0,0001**	0,9852	**<0,0001**	-		
P-MPC1	>0,9999	**<0,0001**	>0,9999	**<0,0001**	0,9982	-	
P-MPC2	>0,9999	**<0,0001**	>0,9999	**<0,0001**	0,9693	0,9997	-

SPP1 day 7	aB-MPC	iB-MPC	fB-MPC	BM-MPC1	BM-MPC2	P-MPC1	P-MPC2
aB-MPC	-						
iB-MPC	0,9999	-					
fB-MPC	0,887	0,7237	-				
BM-MPC1	0,9996	>0,9999	0,676	-			
BM-MPC2	0,9378	0,8098	>0,9999	0,7678	-		
P-MPC1	0,8938	0,7342	>0,9999	0,6869	>0,9999	-	
P-MPC2	0,8793	0,712	>0,9999	0,6638	>0,9999	>0,9999	-

SPP1 day 28	aB-MPC	iB-MPC	fB-MPC	BM-MPC1	BM-MPC2	P-MPC1	P-MPC2
aB-MPC	-						
iB-MPC	**<0,0001**	-					
fB-MPC	**<0,0001**	**<0,0001**	-				
BM-MPC1	**<0,0001**	**<0,0001**	**0,0361**	-			
BM-MPC2	**<0,0001**	**0,0249**	**<0,0001**	0,1471	-		
P-MPC1	**<0,0001**	**<0,0001**	0,9963	**0,0071**	**<0,0001**	-	
P-MPC2	**<0,0001**	**<0,0001**	0,9995	**0,0118**	**<0,0001**	>0,9999	-
